# Direct growth of GaN layer on carbon nanotube-graphene hybrid structure and its application for light emitting diodes

**DOI:** 10.1038/srep07747

**Published:** 2015-01-19

**Authors:** Tae Hoon Seo, Ah Hyun Park, Sungchan Park, Yong Hwan Kim, Gun Hee Lee, Myung Jong Kim, Mun Seok Jeong, Young Hee Lee, Yoon-Bong Hahn, Eun-Kyung Suh

**Affiliations:** 1 Soft Innovative Materials Research Center, Korea Institute of Science and Technology, Jeonbuk 565-905, Republic of Korea; 2 School of Semiconductor and Chemical Engineering & Semiconductor Physics Research Center, Chonbuk National University, Jeonju 561-756, Republic of Korea; 3 Center for Integrated Nanostructure Physics, Institute for Basic Science, Sungkyunkwan University, Suwon, Kyeonggi 440-746, South Korea; 4 Department of Energy Science, Sungkyunkwan University, Suwon, Kyeonggi 440-746, Republic of Korea

## Abstract

We report the growth of high-quality GaN layer on single-walled carbon nanotubes (SWCNTs) and graphene hybrid structure (CGH) as intermediate layer between GaN and sapphire substrate by metal-organic chemical vapor deposition (MOCVD) and fabrication of light emitting diodes (LEDs) using them. The SWCNTs on graphene act as nucleation seeds, resulting in the formation of kink bonds along SWCNTs with the basal plane of the substrate. In the x-ray diffraction, Raman and photoluminescence spectra, high crystalline quality of GaN layer grown on CGH/sapphire was observed due to the reduced threading dislocation and efficient relaxation of residual compressive strain caused by lateral overgrowth process. When applied to the LED structure, the current-voltage characteristics and electroluminescence (EL) performance exhibit that blue LEDs fabricated on CGH/sapphire well-operate at high injection currents and uniformly emit over the whole emission area. We expect that CGH can be applied for the epitaxial growth of GaN on various substrates such as Si and MgO, which can be a great advantage in electrical and thermal properties of optical devices fabricated on them.

Gallium nitride (GaN)-based light emitting diodes (LEDs) have attracted considerable interest for applications such as full color or white LED displays, indoor or outdoor LED lighting, and backlights for liquid-crystal display^1^,^2^,^3^. For commercial use, the development of high quality GaN epilayer for low power consumption LEDs is prerequisite. In general, high-quality GaN layers are synthesized on low-temperature (LT) GaN buffer layer on a single crystal sapphire substrate which has many advantages of high temperature tolerance and hexagonal crystal structure preservation. However, large differences in fundamental properties such as lattice constants and thermal expansion coefficients between GaN layer and sapphire substrate generate high density of threading dislocation (TD) that leads to deterioration of optical and structural properties. With high current injection, GaN-LEDs grown on sapphire substrate seriously suffer from the increasing junction temperature caused by excessive self-heating at the active region due to relatively high operating voltages and poor thermal conductivity of the sapphire substrate that frustrate reliable LED operation, resulting in a large turn-on voltage and a low light output power^4^,^5^. In order to improve reliability and performance of LEDs, most of the current researches focus on two main issues, namely, lowering the TD density and enhancing the thermal dissipation.

Graphene, an ultra-thin two-dimensional form of covalently bonded carbon atoms with a hexagonal lattice structure, has been attracting much attention due to its excellent physical properties such as mechanical flexibility, extremely high intrinsic mobility, high thermal conductivity, high elasticity and high optical transmittance^6^,^7^,^8^,^9^. Accordingly, much efforts have been dedicated to the study and application of graphene-based materials in optoelectronic devices, such as LEDs and solar cells^10^,^11^,^12^,^13^. In particular, graphene has been used as a lateral heat spreader for high power optoelectronic devices and intermediate layer between GaN and sapphire substrate for releasing LED device from sapphire substrate^14^,^15^,^16^. However, direct epitaxial growth of GaN film onto graphene layer on substrates such as sapphire, Si, and MgO is not easily accessible due to the lack of chemical reactivity on graphene which consisted of C-C bond of sp^2^ hexagonally arranged carbon atoms with no dangling bonds^14^.

To resolve this problem, it has been reported that epitaxial growth of GaN film on graphene layers could be achieved by inserting high-density zinc oxide nanowalls as an intermediate layer on plasma-treated graphene^14^,^15^. However, this approach results in low quality GaN layer and high forward voltage of LED devices on them. Han *et al*. introduced patterned graphene oxide as a buffer layer to grow GaN layer on sapphire and shown improved thermal dissipation from LEDs^16^.

In this work, an intermediate layer for the GaN growth on sapphire substrate was constructed by inserting carbon nanotubes and graphene hybrid structure (CGH) which consists of graphene layer scattered with single-walled carbon nanotubes (SWCNTs), to obtain high-quality GaN layers and high efficiency LEDs on them. Optical and structural properties of GaN layer grown on CGH were compared with those of GaN layer directly grown on sapphire in view of luminescence, strain relaxation, TD density, etc. CNTs act as nucleation sites and play a crucial role in the growth of single crystalline high-quality GaN on graphene layer. Also, graphene film acts as a mask, such as SiN_x_ and SiO_2_ of dielectric materials with low surface reactivity for epitaxial lateral overgrowth (ELOG) of GaN layer, which can effectively reduce TD density caused by lattice mismatch between GaN and sapphire substrate. It is also expected that the high thermal conductivity of graphene would endow good heat dissipation for devices fabricated on GaN grown on CGH.

## Results

### Growth of the GaN epilayer on CGH/sapphire

**Figure 1:**
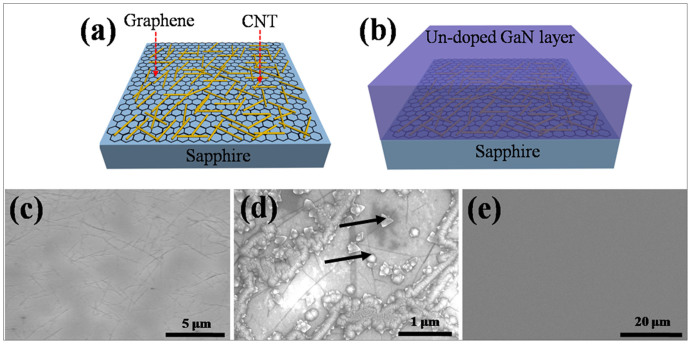
Schematic diagram showing (a) CGH on sapphire, and (b) un-doped GaN layer grown on CGH. SEM images of (c) CGH on sapphire, (d) early stage of GaN buffer layer formed on CGH, and (e) un-doped GaN layer grown on CGH are shown, respectively.

Figs. 1(a) and (b) show schematic diagrams of CGH on sapphire and GaN epilayer grown on CGH/sapphire. Figs. 1(c) – (e) show plan-view scanning electron microscope (SEM) images of CGH on sapphire substrate, initial stage of GaN buffer layer growth on CGH/sapphire, and following un-doped GaN epilayer grown on it, respectively. Diameters and lengths of CNTs are about 1.2 nm and 2–10 µm, respectively. SEM image of CGH reveals that CNTs with random network are well formed on the CVD-synthesized-graphene film. The initial stage of the growth is very important for obtaining epitaxial layers and good quality of resultant film. As can be seen in the SEM image of GaN buffer layer grown on CGH/sapphire at LT in Fig. 1(d), GaN buffer layers start to grow from CNT surroundings and have small islands at other defects formed on graphene film, such as point defects, wrinkles, folds, tears and cracks, as marked by arrows. GaN buffer layers were still not covered on the entire surface at the early growth stage of GaN deposition. During the high temperature GaN growth, this process induces a dramatic morphological change of GaN surface on CGH from 3- to 2- dimensional by ELOG process as shown in Fig. 1(e). ELOG method reduces TD density which will be discussed in detail later. SEM image in Fig. 1(e) shows crack free, mirror-like, and flat surface morphology. We noted that GaN epilayer can also be grown directly on CGH intermediate layer at high temperature without low temperature buffer layer. But the crytallity is much better when low temperature buffer layer is adopted.

**Figure 2:**
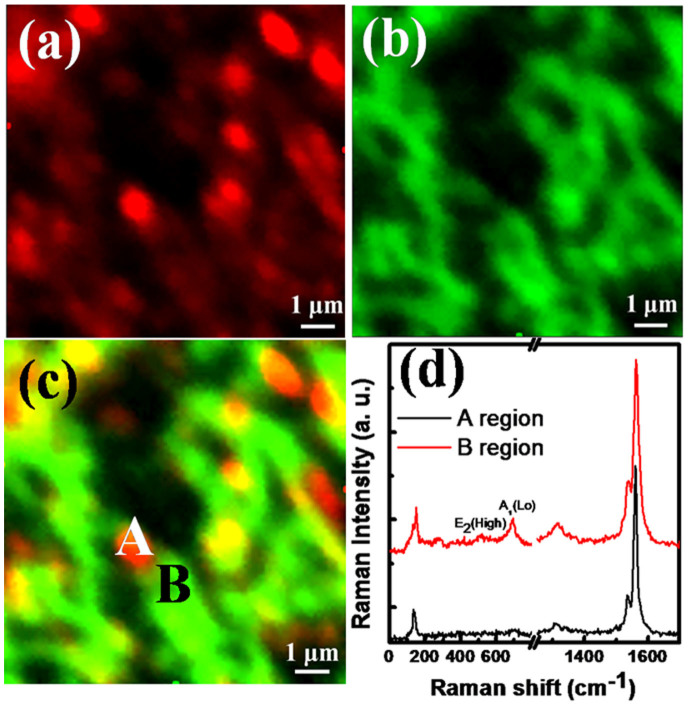
Raman mapping images of GaN buffer layer grown on CGH: (a) radial breathing mode (RBM) of CNT, and (b) A_1_ (LO) mode of GaN. (c) Combination of Raman mapping images shown in Figs. 2(a) and (b). Green parts correspond to A_1_ (LO) mode of GaN while red parts are associated with RBM of CNTs. The overlapped area of RBM and A_1_ (LO) mode are shown in yellow. (d) Raman spectra obtained from regions marked by A and B of Fig. 2(c).

To understand the growth mechanism of GaN buffer layer, we carried out Raman mapping of radial breathing mode (RBM) of CNTs and A_1_ (LO) mode of GaN for 10 × 10 µm^2^ area from the GaN buffer layer on CGH/sapphire and shown in Figs. 2(a) and (b). The RBM, shown in red color in Fig. 2(a), is a unique phonon mode appearing only from single-walled CNTs (SWCNTs) and its observation in the Raman mapping image provides direct evidence that the sample contains SWCNTs. Also, the mapping of A_1_ (LO) mode of GaN in green color, recorded in z(xu)z configuration, is in congruent with that of RBM of SWCNTs in positions, which is a proof for the formation of GaN layer on CGH, as can be seen in Fig. 2(b). Fig. 2(c) shows the images of Figs. 2(a) and (b) overlapped each other. Green parts, corresponding to A_1_ (LO) mode of GaN, are observed around red parts associated with RBM of SWCNTs. The overlapped area of RBM and A_1_ (LO) mode are manifested in yellow. In Fig. 2(d), we show Raman spectra measured from positions A and B marked in Fig. 2(c), respectively. Two major peaks associated with the RBM and G-band of SWCNTs are observed from both A and B region. In addition, two prominent peaks are observed in the Raman spectrum from position B: E_2_ (high) and A_1_ (LO) modes of GaN. The Raman mapping clearly demonstrates that the GaN buffer layer grows in the vicinity of SWCNTs, neither on the SWCNTs nor on normal graphene layer at the initial stage of the growth, in agreement with the SEM image of Fig. 1(d).

### Surface morphology and crystalline quality of the GaN epilayer grown on CGH/sapphire

**Figure 3:**
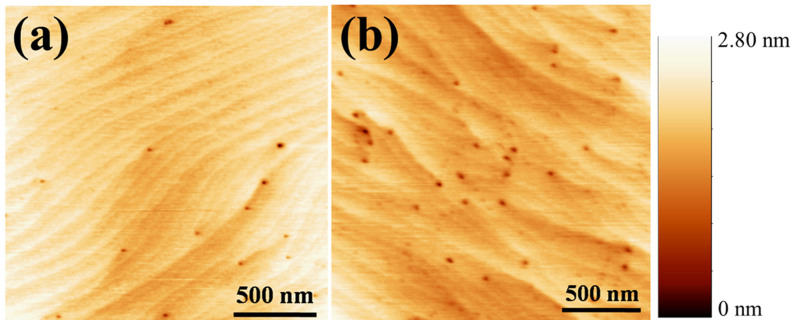
AFM images of the surface of GaN epilayers grown on (a) CGH, and (b) sapphire, respectively.

In order to investigate the dislocation pit density and surface morphology of the GaN layer grown on CGH/sapphire, as shown in Figs. 3(a) and (b), atomic force microscope (AFM) measurement is carried out for 2 × 2 µm^2^ area, and compared with those of conventional GaN grown on sapphire. The root mean square (RMS) values for the surface roughness of the GaN layer grown on CGH/sapphire and on sapphire are approximately 0.16 and 0.21 nm, respectively. From the well-arranged steps and terraces, it is clear that the 2D step growth mode is dominant over the entire substrate. However, the AFM images of both the GaN layer grown on CGH/sapphire and on sapphire shows a few pits positioned at the surface step. These pits correspond to the surface termination of pure-screw- or mixed-screw-edge dislocations propagating to the GaN surface. Etch-pit densities of GaN layer grown on CGH/sapphire and on sapphire are 8.3×10^8^ cm^−2^ and 2.5×10^9^ cm^−2^, respectively, showing reduced etch-pit density in GaN on CGH/sapphire due to the suppression of threading dislocation propagation during the ELOG process^17^. These results imply that the high-quality GaN layer can be achieved by inserting CGH layer before the growth of GaN layer.

**Figure 4:**
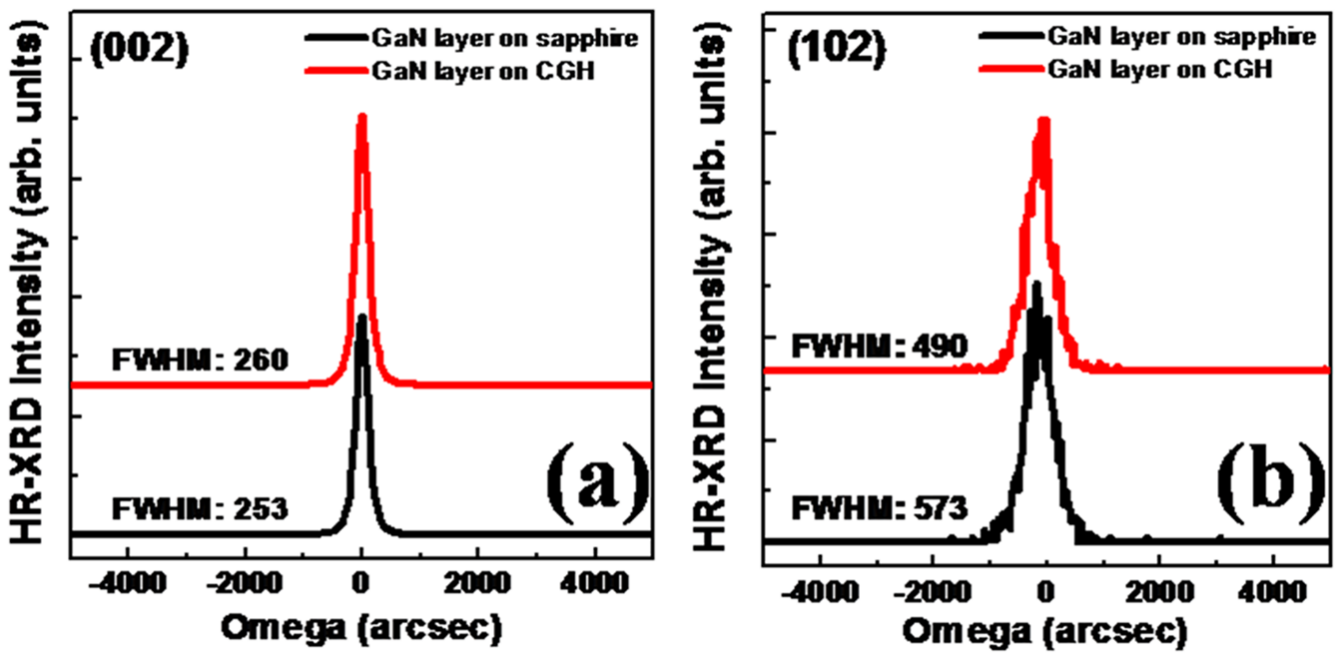
XRD omega rocking curves of (a) the symmetrical (002) and (b) asymmetrical (102) reflection of GaN layer grown on CGH/sapphire and on sapphire, respectively.

To compare the crystalline quality of GaN layer grown on CGH/sapphire with that on sapphire, high resolution x-ray diffraction (HR-XRD) rocking curves (ω-scans) of the (002) symmetry and (102) asymmetry reflections are measured and shown in Figs. 4(a) and (b). The full width at half-maximum (FWHM) values for GaN layer grown on CGH/sapphire and on sapphire in the (002) reflection are found to be 260 and 253 arcsec, respectively, showing similar values. In the case of an asymmetrical (102) reflections, FWHM values of GaN layer with and without CGH are observed 490 and 573 arcsec, respectively. It is well known that the FWHM of XRD in the (102) reflection corresponds to the lattice distortion from all components of the TDs including pure edge, screw and mixed screw-edge dislocations, while the FWHM of (002) reflection is associated with screw and mixed screw-edge dislocations^18^. Even though the value of FWHM for GaN layer grown on CGH/sapphire in the (002) reflection is similar to that of GaN layer on sapphire, the FWHM of GaN layer with CGH in the (102) reflection is much lower than that of conventional GaN layer without CGH. This indicates the reduced pure edge dislocation owing to the ELOG process. FWHM values obtained from GaN layer on CGH/sapphire are similar to those previously observed from GaN layers grown by ELOG using nanometer-sized masks of thin SiN_x_ inserting layer^19^. We point out that the GaN growth condition should be adjusted for different substrates, and the crystallinity can be further improved by optimization of the growth condition for GaN on CGH/sapphire in the respective growth system.

### Strain relaxation and improved optical property in GaN on CGH/sapphire

**Figure 5:**
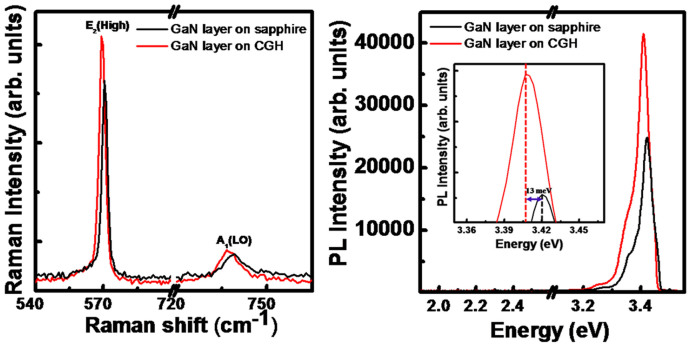
(a) Raman and (b) PL spectra of GaN layer grown on CGH/sapphire and on sapphire, respectively, recorded at room temperature.

Fig. 5(a) shows micro-Raman spectra of GaN layer grown on CGH/sapphire and on sapphire, respectively. Two prominent peaks associated with E_2_ (high) and A_1_ (LO) modes of GaN layer grown on CGH/sapphire and on sapphire are observed in the z(xu)z configuration. The FWHM values of the E_2_ (high) peak by Lorentzian function fittings are obtained to be 3.12 and 3.28 cm^−1^ for GaN epilayer grown with and without CGH, respectively, indicating the improvement of the crystalline quality caused by the reduced TD in GaN with CGH intermediate layer. The E_2_ (high) mode is particularly sensitive to the biaxial strain in GaN layer. In general, GaN layer grown on sapphire substrate faces compressive strain. Frequencies of the E_2_ (high) mode are found to be 569.0 and 570.4 cm^−1^ for GaN layer on CGH/sapphire and on sapphire, respectively. The biaxial stress can be calculated, according to Δ*ω* = *Kσ_xx_*, from the measured Raman frequency shift of a given E_2_ (high) phonon mode if the linear stress coefficient *K* is known^20^. We adopt the theoretical value, 2.56 cm^−1^/GPa^20^, for the stress coefficient and a standard frequency value 567.6 cm^−1^ of the unstrained bulk GaN for the E_2_ (high) mode^21^. The calculated residual stresses in the GaN layer with and without CGH intermediate layer are 0.54 and 1.10 GPa, respectively, indicating that GaN film grown on CGH/sapphire has lower degree of residual stress than that of GaN layer grown on sapphire substrate. This reflects the efficient relaxation of the residual stress in the GaN layer by ELOG process of GaN layer on CGH/sapphire. Also, the A_1_ (LO) phonon mode of GaN film on CGH/sapphire, which corresponds to the atomic vibration along the c-direction, was shifted toward the low frequency, 736.7 cm^−1^, from 739.5 cm^−1^ of GaN on sapphire, due to the combined influence of phonon deformation potential and elastic constants^22^.

The effect of CGH on optical properties of GaN layer is studied by photoluminescence (PL) as shown in Fig. 5(b). The PL spectra of the GaN layer on CGH/sapphire and on sapphire are dominated by near band-edge (NBE) luminescence at 3.407 and 3.420 eV, respectively. It is well known that the NBE intensity is sensitive to the defect density^23^. The PL intensity of GaN layer on CGH/sapphire is significantly increased, by about 170%, compared to that of GaN layer on sapphire, indicating the improved crystalline quality by the reduced defect density with TD, in agreement with the AFM image of Fig. 3(b). Yellow luminescence (YL) band at 2.2 ~ 2.3 eV which is one of the most direct factors to reflect the defects in GaN layer is not observed in both samples. The NBE luminescence for GaN layer on CGH/sapphire is red-shifted by about 13 meV with respect to that observed from GaN layer on sapphire. Taking into account the biaxial compressive stress-dependent shift of the NBE luminescence peak position at room temperature, i. e., 23 meV/GPa^24^, the difference in the degree of stress between GaN layers on CGH/sapphire and on sapphire is calculated to be 0.56 GPa, in good agreement with the value obtained from Raman spectra. This red-shift confirms the efficient relaxation of the residual compressive strain in the GaN layer grown on CGH/sapphire.

### Electrical and optical characteristics of LEDs fabricated on GaN with CGH interlayer

**Figure 6:**
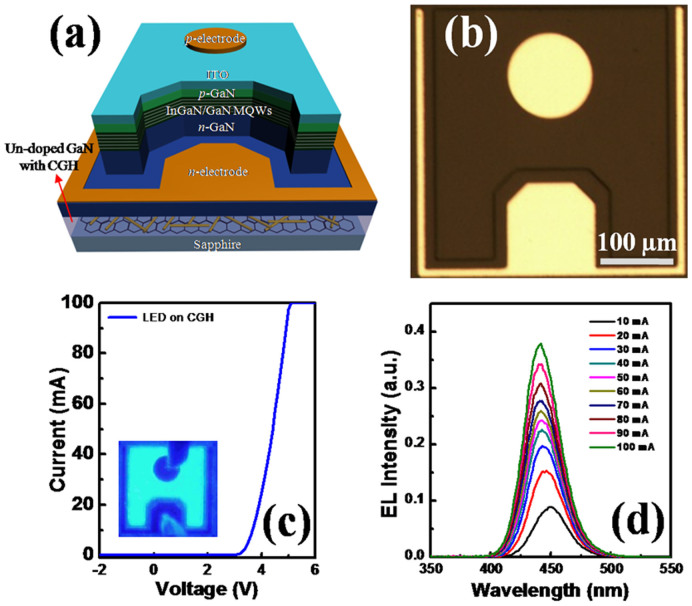
(a) Schematic diagram, (b) optical image, (c) I-V curve of fabricated InGaN/GaN MQWs LED on CGH/sapphire. (d) EL spectra as a function of current in the LED on CGH/sapphire. The inset of Fig. 6 (c) is its EL image at an injection current of 20 mA.

We adopted the high-quality GaN layer on CGH for the fabrication of blue LEDs. A schematic diagram and optical image of fabricated LEDs with GaN grown on CGH intermediate layer are shown in Figs. 6(a) and (b). LEDs with CGH present a rectifying behavior without showing a reverse bias leakage current. The forward voltage at an injection current of 20 mA is 3.8 V, as shown in Fig. 6(c). Also, a bright and uniform light emission in the electroluminescence (EL) image (inset) is observed over the whole surface of the LED. Fig. 6(d) shows the EL spectra of LED with CGH as a function of the injection current from 10 to 100 mA. Our LED with CGH operates well at injection currents up to 100 mA examined in this work. The EL emission wavelength has shifted from 449 nm at 10 mA to 442 nm at 100 mA. The blue-shift of EL peak position with increasing injection current is due to the band filling effect of the localized energy states formed by potential fluctuation in MQWs and the screening effect of the polarization-induced electric field, as was observed in previous report^25^.

**Figure 7:**
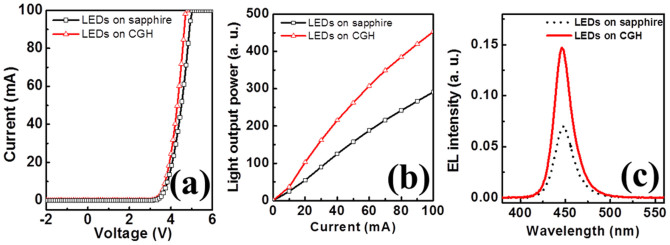
(a) The I-V characteristics, (b) L-I curves as a function of current and (c) EL spectra at injection current of 20 mA for LEDs on CGH/sapphire and on sapphire, respectively.

For comparison, we present the opto-electircal characteristics of our LEDs with and without CGH intermediate layer in Figs. 7(a)–(c). The I-V characteristics of InGaN/GaN MQWs LEDs on sapphire and on CGH/sapphire are shown in Fig. 7 (a). The forward voltages at an input current of 20 mA are found to be 3.80 and 3.86 V for LEDs with and without CGH, respectively. Figures 7(b) and (c) show L-I curves and EL spectra for fabricated LEDs with and without CGH, respectively. The emission wavelengths of LEDs on CGH and on sapphire were 446 and 449 nm, respectively. The light output powers and EL intensity for LEDs with CGH intermediate layer was increased remarkably compared to those of LEDs on sapphire. We suggest the mechanism of the efficiency enhancement associated with CGH is due to the increased IQE caused by the reduction of threading dislocation density, and enhanced light extraction by geometrical shape of carbon nanotubes which efficiently scatter the guided light to find escape cones.

## Discussion

In the growth of hetero-epitaxial layers, the film growth mode is substantially governed by the elastic strain energy which increases with the layer thickness and the strain induced by the lattice mismatch. Therefore, for the growth of high quality films it is essential to release the strain energy accumulated in the growing film. It is known that the elastic energy can be released by plastic relaxation, i.e. dislocation formation^26^. In this work, the SWCNTs on graphene act as dislocation centers, resulting in the formation of kink bands along SWCNTs with the basal plane of the substrate, and play the role of nucleation seeds.

It is reported that the deformation such as kinking and buckling can be induced by the generation and glide of dislocations along the basal planes without the activation of additional non-basal slip systems and the kink-band formation occurs in grains oriented with the basal planes^27^,^28^. Therefore, the SWCNTs-induced kink-bands and steps provide nucleation sites and trigger the formation of GaN nuclei along the SWCNTs. This explanation is supported by the SEM observation of the initial stage of GaN buffer layer growth (see Fig. 1(d)). The observation also suggests a mechanism by which the kink bands might grow and propagate along SWCNTs. This growth mechanism is also supported by the Raman mapping image and spectrum, as shown in Figs. 2(c) and (d) which exhibit E_2_ (high) and A_l_ (LO) mode corresponding to GaN around SWCNTs. After the initial stage of GaN buffer layers, the growth mode is changed to layer-by-layer growth, i.e., Frank-van der Merwe, to form high quality ELOG films as shown in Fig. 1(e).

In summary, to obtain high-quality GaN layer, we propose to adopt SWCNTs and graphene film hybrid structure as an intermediate layer between GaN layer and sapphire substrate. The improvement of crystalline quality and the relaxation of residual compressive stress in GaN layer grown on CGH/sapphire compared to those of GaN layer on sapphire were confirmed by Raman scattering, PL and XRD spectra; this is because the epilayer growth can be achieved through the ELOG process occurred during the growth of GaN on CGH intermediate layer. The current-voltage characteristics and electroluminescence performance show that LEDs fabricated on GaN layer grown on CGH/sapphire well-operate at the high injection current and uniformly emit over the whole emission area. We suggest that the use of CGH for the growth of GaN opens up the possibility of GaN growth on various substrates other than sapphire, such as Si, for extended performance of III-nitride LEDs. Furthermore, the advantageous thermal properties of graphene and CNTs can improve the thermal characteristics of devices fabricated on GaN grown on CGH intermediate layer.

## Methods

### Synthesis of graphene and transfer to sapphire substrate

Large scale graphene layers were synthesized on ~35 µm thick Cu-foil (Nippon Mining Corp.) by chemical vapor deposition (CVD) method. The surface of synthesized-graphene on Cu-foil forms uniform monolayer because of low carbon solubility in copper. Details can be found in Ref. 29. Prior to the buffer layer growth, CVD-grown graphene film was transferred onto the flat sapphire substrate using poly methyl methacrylate (PMMA). To prevent the oxidation of the substrate and remove the PMMA, the substrate was annealed under a gas flow of rate H_2_:Ar = 90:10 sccm for 30 min at 500°C.

### Formation of SWCNTs on graphene

Arc-SWCNTs (Single Walled Carbon Nanotubes purchased from Nanosolution Corp.) were purified by thermal and acid treatment procedures, and dispersed in water using sodium dodecyl sulfate (SDS) surfactant. Subsequently, the SWCNTs were spin-coated at 4000 rpm for 10 seconds onto the graphene film on c-plane sapphire substrates.

### Growth of GaN layer on CGH/sapphire

The GaN epilayer was grown on CGH on sapphire substrate by metal-organic chemical vapor deposition (MOCVD). It should be noted that the growth condition of GaN layer on CGH/sapphire should be different from that of GaN layer grown directly on sapphire substrate to grow epilayer. For conventional GaN growth, a 20 nm-thick GaN buffer layer was deposited on sapphire substrate at 560°C for 100 s under a growth pressure of 400 mbar before the growth of an un-doped GaN layer at 1100°C under 400 mbar. However, to grow GaN layer on the CGH/sapphire, the LT nucleation layer was deposited on CGH/sapphire at 560°C for 5 min under a growth pressure of 635 mbar and the growth of a 3 µm-thick un-doped GaN layer was followed at 1130°C for 2 h under a growth pressure of 100 mbar.

### Growth and fabrication of blue-LED structures

LED structure is consisted of an un-doped GaN layer, a Si-doped *n*-type GaN layer, five-pairs of InGaN/GaN MQWs, and a Mg-doped *p*-type GaN layer. A Si doped *n*-GaN layer with a thickness of 2-µm was grown on un-doped GaN layer on CGH/sapphire at 1100°C and 400 mbar for 60 min. Then, for active layers, five pairs of InGaN quantum wells and GaN barrier layers with thicknesses of 3 and 12 nm, respectively, were grown at 720°C and 810°C, respectively. Finally, 150 nm-thick *p*-type GaN epilayer was grown at 980°C. After the growth of LED wafer, discrete LED devices were fabricated with a chip size of 350 × 350 µm^2^ in which the mesa region was defined by an inductively coupled plasma (ICP) etcher using Cl_2_/BCl_3_/Ar gases until *n*-GaN layer was exposed for *n*-electrode contact. Subsequently, 200 nm-thick indium tin oxide (ITO) layer as transparent and current spreading electrode was formed on *p*-GaN layer by electron beam evaporator. As a final step, Cr (50 nm)/Au (250 nm) metals for the *p*- as well as the *n*-electrode were deposited onto both the ITO and the *n*-GaN layer using electron beam evaporator.

### Characterization

Field emission scanning electron microscopy (FESEM, Hitachi S-4800) was used to observe the surface morphology of CGH on sapphire substrate, initial stage of GaN buffer layer on CGH/sapphire, and the following un-doped GaN epilayer grown on them, respectively. To understand the growth mechanism of GaN buffer layer, Raman mapping measurements (NT-MDT, Russia) of RBM of CNT and A_1_ (LO) mode of GaN from the GaN buffer layer on CGH/sapphire were performed. The surface topography of GaN layer on CGH/sapphire and on sapphire was probed by atomic force microscope (AFM, Digital Instruments, Nanoscope IV A) in tapping mode. Both samples of GaN layer grown with and without CGH were measured by X-ray diffraction (XRD, X'pert-MRD) with scan rate of 2 degree/min from 20 to 80 degree to compare the crystalline quality. Raman spectroscopy (Renishaw) using 514 nm-line of an Ar-ion laser and photoluminescence (PL) with a 325 nm-line of a He-Cd laser were used to examine the crystalline quality and the residual strain of GaN layer grown on sapphire and on CGH/sapphire. Current-Voltage (I-V) and electroluminescence (EL) measurements on the LED chips were carried out using a probe station system.

## Author Contributions

T.H.S. and E.-K.S. planned and designed experiments and analysis, and prepared the manuscript. S.P. and M.J.K. carried out the synthesis of graphene and transfer to sapphire substrate. T.H.S. and A.H.P. performed GaN growth by MOCVD and fabricated the light emitting diodes. The device characterization was done by T.H.S. and G.H.L. The Raman measurement was carried out by Y.H.K., M.S.J. and Y.H.L. Y.-B.H. contributed to clarity the growth mechanism. All authors discussed the results and implications and commented on the manuscript at all stages.

## Supplementary Material

Supplementary Information Supplementary information
