# Supplementary material for: Direct growth of GaN layer on carbon nanotube-graphene hybrid structure and its application for light emitting diodes
Source: Sci Rep. 2015 Jan 19;5:7747. doi: 10.1038/srep07747 (PMC4297988; doi:10.1038/srep07747)
Supplement: Supplementary Information [file srep07747-s1.pdf]

Supplementary Information

Direct growth of GaN layer on CNT-graphene hybrid structure and its application for light emitting diode

Tae Hoon Seo,[1] Ah Hyun Park,[2] Sungchan Park,[1] Yong Hwan Kim,[3] Gun Hee Lee,[2] Myung Jong Kim,[1] Mun Seok Jeong,[3] Young Hee Lee,[3] Yoon-Bong Hahn,[2] and Eun-Kyung Suh[2,]*

[1] Soft Innovative Materials Research Center, Korea Institute of Science and Technology, Jeonbuk 565-905, Republic of Korea

[2] School of Semiconductor and Chemical Engineering & Semiconductor Physics Research Center, Chonbuk National University, Jeonju 561-756, Republic of Korea

[3] Center for Integrated Nanostructure Physics, Institute for Basic Science, Sungkyunkwan University, Suwon, Kyeonggi 440-746, South Korea and department of Energy Science, Sungkyunkwan University, Suwon, Kyeonggi 440-746, Republic of Korea

* **Corresponding author**. Tel.: +82 63 2703606; fax: +82 63 2703585

E-mail: eksuh@jbnu.ac.kr (E. K. Suh)

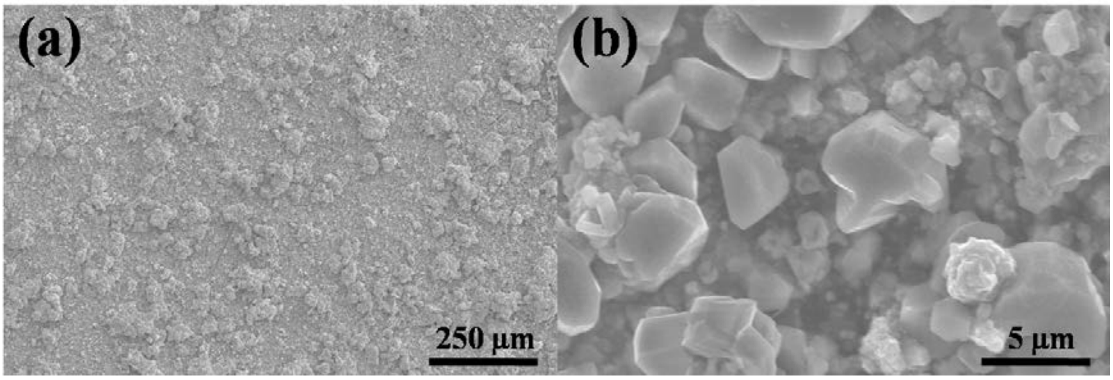

Figure S1. (a) Low and (b) high resolution SEM images of GaN layer grown on graphene/sapphire. Only micro-crytal but no epitaxial layer growth is achieved.

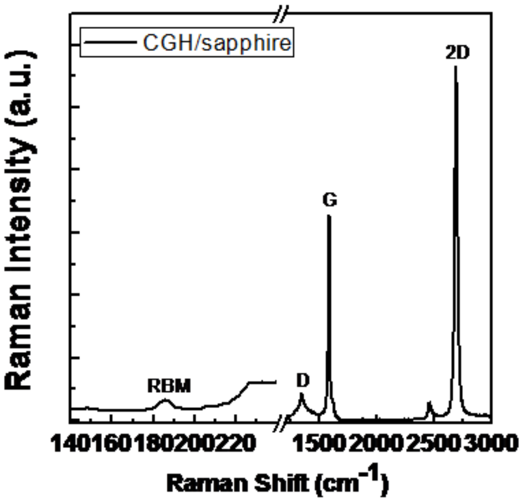

Figure S2. Raman spectrum of CGH on sapphire. Raman peaks associated with SWCNTs and graphene layer are observed.
